# Polycomb repressive complex 2 and its core component EZH2: potential targeted therapeutic strategies for head and neck squamous cell carcinoma

**DOI:** 10.1186/s13148-024-01666-2

**Published:** 2024-04-10

**Authors:** Yuxi Cheng, Zhengzheng Song, Xiaodan Fang, Zhangui Tang

**Affiliations:** 1 Xiangya Stomatological Hospital and Xiangya School of Stomatology, Central South University, Changsha, 410008 Hunan China; 2 Clinical Research Center of Oral Major Diseases and Oral Health & Academician, Central South University, Changsha, 410008 Hunan China

**Keywords:** Head and neck squamous cell carcinoma, PRC2, EZH2, Polycomb repressive complex, Transcription regulation, EMT, Targeted therapy

## Abstract

The polycomb group (PcG) comprises a set of proteins that exert epigenetic regulatory effects and play crucial roles in diverse biological processes, ranging from pluripotency and development to carcinogenesis. Among these proteins, enhancer of zeste homolog 2 (EZH2) stands out as a catalytic component of polycomb repressive complex 2 (PRC2), which plays a role in regulating the expression of homologous (Hox) genes and initial stages of x chromosome inactivation. In numerous human cancers, including head and neck squamous cell carcinoma (HNSCC), EZH2 is frequently overexpressed or activated and has been identified as a negative prognostic factor. Notably, EZH2 emerges as a significant gene involved in regulating the STAT3/HOTAIR axis, influencing HNSCC proliferation, differentiation, and promoting metastasis by modulating related oncogenes in oral cancer. Currently, various small molecule compounds have been developed as inhibitors specifically targeting EZH2 and have gained approval for treating refractory tumors. In this review, we delve into the epigenetic regulation mediated by EZH2/PRC2 in HNSCC, with a specific focus on exploring the potential roles and mechanisms of EZH2, its crucial contribution to targeted drug therapy, and its association with cancer markers and epithelial–mesenchymal transition. Furthermore, we aim to unravel its potential as a therapeutic strategy for oral squamous cell carcinoma.

## Introduction

Although the dominant belief indicates that cancer is caused by numerous genetic mutations, extensive pan-cancer analyses have indicated that about 5% of cancer cases do not have identifiable driver mutations that can explain the formation of tumors. This discovery suggests that genetics alone may not explain all aspects of cancer development. Instead, non-genetic changes seem to offer an alternative pathway for the progression, metastasis, and development of drug resistance in cancer cells. For instance, in pancreatic ductal adenocarcinoma, metastasis is not primarily driven by gene mutations, but rather by significant epigenetic reprogramming, highlighting the prevalent involvement of epigenetic modifications in this context [1]. Furthermore, ependymoma, a childhood brain tumor, exhibits an unusually low mutation rate, suggesting that cancer emerges through the intricate interplay between genetic and non-genetic processes that generate and evolve the disease [2]. From a broader perspective, cancer can be viewed as an "epigenetic disease," underscoring the importance of dysregulation as a pivotal driving force in tumor development.

Human HNSCC is a prevalent form of cancer, with around 550,000 new cases being diagnosed annually worldwide. This disease is characterized by rapid cell division, the tendency to spread to nearby lymph nodes, and a generally unfavorable prognosis [3, 4]. Unfortunately, there are limited treatment options available for HNSCC, particularly in terms of targeted therapies. Consequently, finding effective and efficient treatment strategies for patients with HNSCC remains an urgent challenge.

The PcG proteins have been implicated in a wide range of biological processes, and their significance in cancer research is becoming increasingly evident. Among the PcG proteins, EZH2 is a key player as one of the three core subunits of the PRC2. Multiple studies have suggested that EZH2 mutations or overexpression occur in various hematological cancers and solid tumors, including HNSCC.

Notably, EZH2 has been found to be highly upregulated in neuroendocrine prostate cancer, making it an attractive target for therapy, especially in combination with high-affinity androgen receptor pathway inhibitors. To inhibit EZH2's enzymatic activity, several small molecule inhibitors have been developed. While these inhibitors have shown promising efficacy in lymphomas, their effectiveness against HNSCC remains largely understudied. Additionally, the histone methyltransferase activity (MTase) of EZH2 holds promise as a potential treatment strategy for HNSCC [5]. Research in this area is eagerly anticipated. Moreover, EZH2 may serve not only as a biomarker for predicting prognosis, targeting EZH2 in HNSCC not only holds promise as a personalized therapeutic approach for patients but also requires further exploration to completely grasp the clinical implications it may have.

## Polycomb repressive complex 2

The discovery of the polycomb repressor complexes PRC2 and PRC1 can be traced back to genetic studies conducted on Drosophila, which shed light on the existence of polycomb subunits [6]. Subsequently, it was revealed that mammalian transcription is intricately regulated by chromatin dynamics, ultimately giving rise to diverse phenotypes. Although sequence-specific transcription factors play a crucial role in orchestrating accurate transcriptional programs, their function is often intertwined with that of chromatin modifiers and remodelers. Together, these molecular players work in harmony to ensure proper regulation of gene expression.

In embryonic mammalian cells, the promoters of developmental transcription factors in mouse embryonic stem cells (ESCs) exhibit a unique chromatin landscape marked by the simultaneous presence of trimethylated histone H3 lysine 4 (H3K4me3) and trimethylated histone H3 lysine 27 (H3K27me3). This phenomenon, known as "bivalence," plays a crucial role in regulating gene expression during early embryonic cell differentiation. H3K4me3 promotes transcription by facilitating Trithorax complex accumulation, whereas H3K27me3, associated with polycomb complex accumulation, acts to suppress gene expression [7, 8]. The study has revealed the functional antagonism between the PcG and Trithorax Group (TrxG) protein families. PcG proteins play a role in maintaining gene transcriptional inhibition by facilitating chromatin compaction, while TrxG proteins maintain transcriptional permission through antagonistic inhibition. Mechanistically, TrxG proteins counteract the inhibitory effect of chromatin compaction mediated by PcG proteins, thereby allowing transcriptional activation. This de-inhibitory activity is achieved through the action of two types of complexes: (1) Compass-like methyltransferase/demethylase complexes, and (2) ATP-dependent SWI/SNF chromatin remodeling complexes. Although the precise mechanism by which TrxG proteins counteract PcG-mediated inhibition is still under intensive investigation, mounting evidence suggests that TrxG proteins achieve this by selectively displacing PcG proteins from specific sites. Meanwhile, recent studies have revealed a new finding that contradicts previous research: the co-activation of trx and PRC2 genes actually leads to a synergistic effect in the tumor environment. Previous findings suggested that inhibiting EZH2 could counteract tumors caused by the inactivation of another TrxG complex, SWI/SNF or MLL3 complex. However, inhibiting EZH2 as a treatment for tumors with MLL3 or SWI/SNF gene alterations may not be applicable to tumors with MLL1/2-Compass-like gene inactivation or inhibition. This phenomenon can be explained by two mechanisms. Firstly, the co-activation of trx and PRC2 genes can rescue the inhibition of Hox and Gadd45 genes induced by PRC2 gene knockdown, which aligns with the expectations of typical PcG/trxG targets [9, 10]. The bivalent state is not merely a result of cell culture conditions or cellular heterogeneity. It accounts for the lineage-specific expression of developmental transcription factor genes during early embryonic cell differentiation [11]. The PcG (polycomb group) proteins, in particular, play a significant role in gene silencing. They function by mediating chromatin compaction and are critical for maintaining repression of PcG target genes. Additionally, the participation of PcG proteins in the three-dimensional (3D) organization of the genome plays an active role in the overall regulation of gene expression [12–14].

Function of PcG Proteins in Transcription Activation: The independent recruitment of PRC1 by H3K27me3 is considered a crucial factor that enhances the transcriptional activity of PcG proteins. PcG proteins exert their influence on chromatin and form two distinct complexes with different enzymatic activities [15]. PRC1 and PRC2 are not single-formed complexes but a collection of multiple mutually exclusive complexes. The core of PRC1 primarily consists of really interesting new gene 1A and 1B (RING1A/RING1B), while the core of PRC2 mainly comprises embryonic ectoderm development (EED), retinoblastoma binding protein 4/7 (RBBP4/7), and enhancer of zeste 1/2 (EZH1/2) [16].

PRC1, a histone ubiquitin ligase with E3 ubiquitin ligase activity, acts on specific residues (Lys118 and Lys119) of histone H2A in mammalian cells. It primarily monoubiquitinates these residues and promotes local chromatin compaction. However, recent investigations have unveiled additional transcriptional activation effects of PRC1. Moreover, research on higher-order chromatin structures has identified a novel role of PRC1 in facilitating distant interactions [17, 18]. It is important to note that PRC1 is not a single complex but consists of different complexes characterized by subunit classifications, each conferring distinct biological functions [19]. Over the past decade, extensive research has uncovered the remarkable complexity of mammalian PRC1 complexes, which represents a crucial advancement in understanding the diverse functional implications of these essential epigenetic regulators. A study conducted in HEK293 cells utilized affinity purification and mass spectrometry to not only confirm the presence of known complexes but also discover several new PRC1 complexes. One interesting finding from this research is the classification of mammalian PRC1 complexes into six groups (PCGF1-6) based on their exclusive association with one of the six PCGF proteins. RING1A or RING1B, which are analogous to Drosophila Sce/dRing, are shared components across all six groups and are referred to as PRC1.1–6 depending on the associated PCGF protein. PRC1.2/4 consists of different Pc counterparts (CBX2/4/6/8), three proteins with multiple homeobox domains (PHC1-3), and small amounts of Scm counterparts (SCMH1, SCML1, and SCML2). These complexes, due to their composition resembling the original Drosophila PRC1 complex and the presence of CBX/PHC/Scm, are referred to as canonical PRC1 complexes (cPRC1) [20–23]. For example, PRC1.5 comprises components such as Autism Susceptibility Candidate 2 (AUTS2), which recruits CK2 and p300. These components inhibit the E3 ubiquitin ligase activity through phosphorylation of RING1B and promote transcription by adding acetylation to histone tails [24–26].

**Fig. 1:**
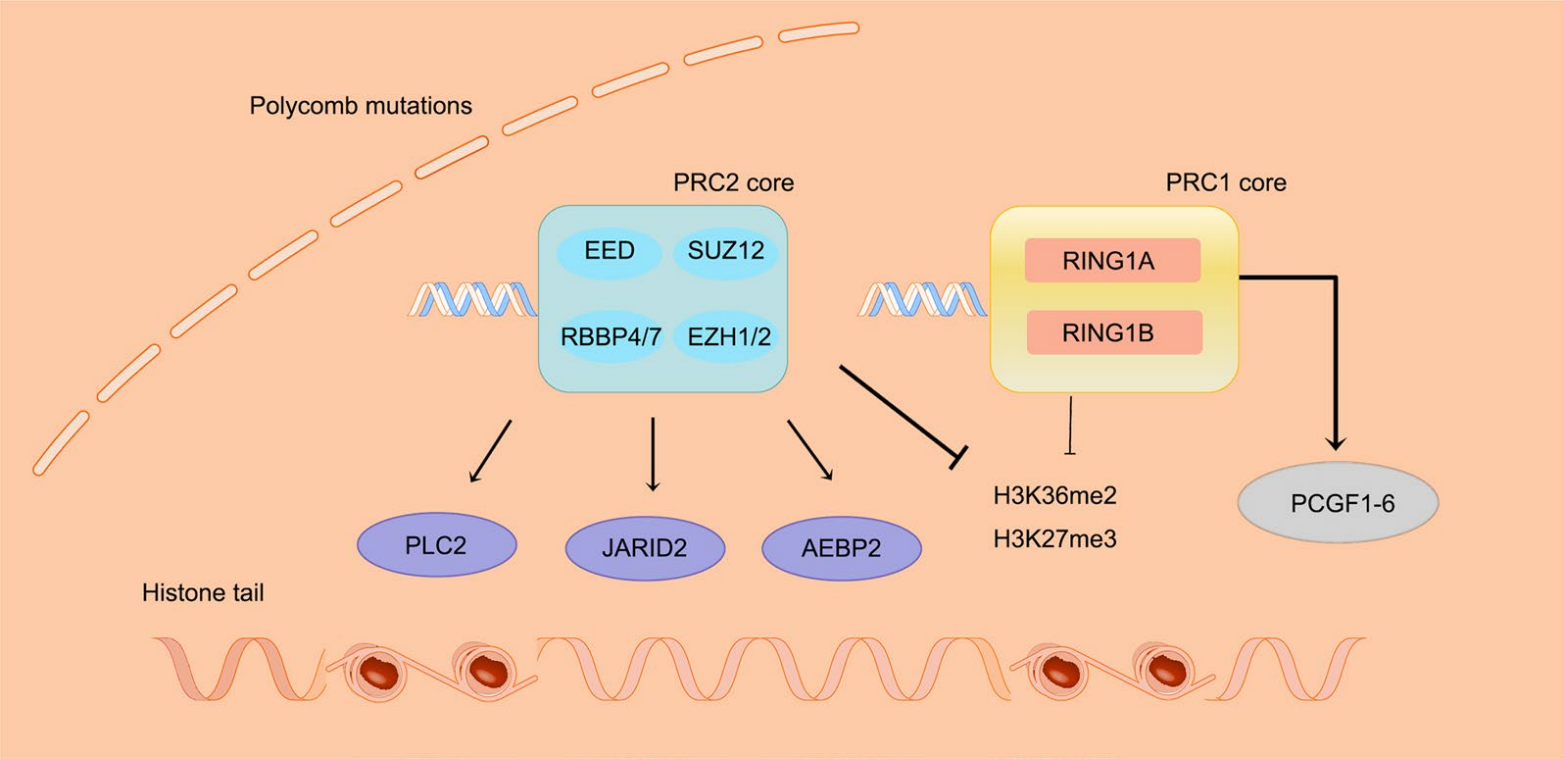
The mechanism of epigenetic regulation of PcG. The core of PRC1 primarily consists of really interesting new gene 1A and 1B (RING1A/RING1B), while the core of PRC2 mainly comprises embryonic ectoderm development (EED), retinoblastoma binding protein 4/7 (RBBP4/7), and EZH1/2. PRC2 deposits trimethylation of H3K27me3 to genomic loci. Then, related proteins drive cPRC1 to PRC2 pre-occupied loci and deposit monoubiquitylation of histone H2A at lysine 119 H2AK119ub1 [109]

PRC2 is a histone methyltransferase. PRC2, unlike PRC1, relies solely on the presence and integrity of its four core subunits for its catalytic activity (Fig. 1). The enzymatic activity of PRC2, along with its interactions with cofactors and DNA elements, is under strict regulation. Recent studies have revealed the existence of additional auxiliary proteins and the characterization of two distinct subtypes of PRC2 complexes, namely PRC2.1 and PRC2.2. PRC2.1 includes polycomb-like protein 1 (PCL1, PCL2, PCL3) and EPOP or PALI1, in addition to the core subunits. On the other hand, PRC2.2 specifically consists of highly conserved JARID2 and AEBP2 on human chromosomes [27–29]. Various effectors, including typical PRC1 and proteins containing the BAH module, recognize the histone H3K27me3 catalyzed by PRC2. These effectors contribute to gene silencing through different mechanisms, such as chromatin compaction associated with phase separation and histone deacetylation. Among these complexes, PRC2.1 includes polycomb-like proteins, which are primarily involved in recruiting PRC2 to CpG islands (CGIs), regions that are enriched with CpG dinucleotides. On the other hand, PRC2.2 contains JARID2, responsible for binding to the monoubiquitinated histone H2AK119ub [29, 30].

## EZH2 epigenetic regulatory elements

**Fig. 2:**
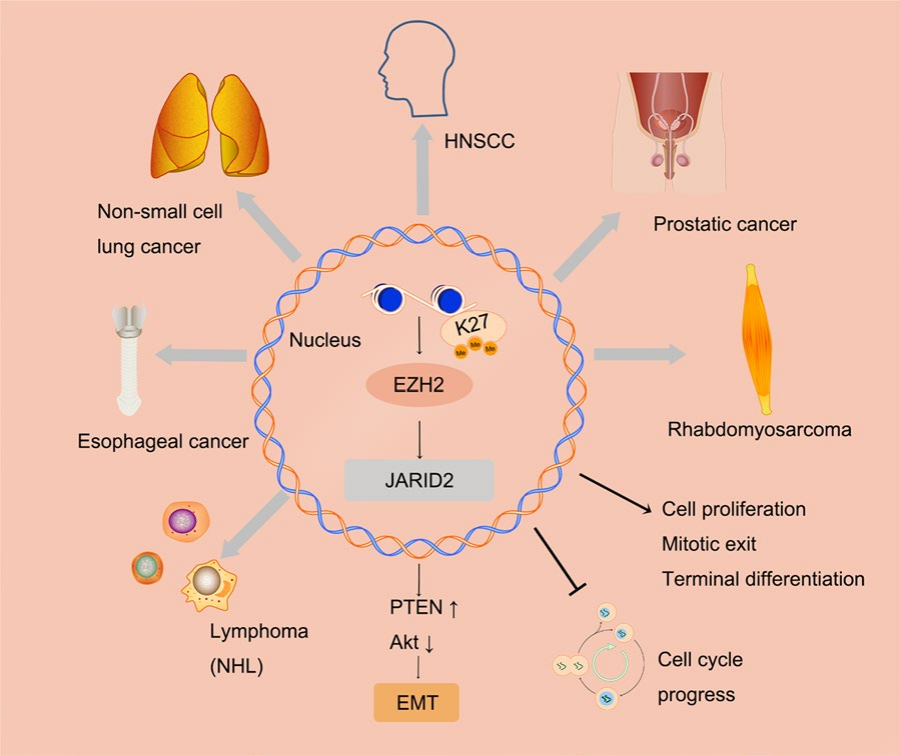
The epigenetic regulation of PRC2/EZH2 occurs in various tumors. In recent years, there have been reports of frequent overexpression of EZH2 in various human epithelial tumors, such as prostate cancer, breast cancer, gastric cancer, non-small cell lung cancer, esophageal cancer, melanoma, oral cancer, and endometrial cancer. Moreover, its overexpression has been correlated with enhanced tumor cell proliferation and unfavorable prognosis [65, 111]

In mouse ESCs, EZH2 functions as the main methyltransferase for H3K27me3, while other core members of the complex play roles in stability, recruitment, protein–protein interactions, and allosteric activation. Extensive research and problem-solving efforts have been dedicated to EZH2 within the context of PRC2. Acting as the primary catalytic subunit of the PRC2 complex, EZH2 depletion or inhibition leads to a near-total loss of H3K27me2/3 marks [31, 32]. In contrast, EZH1 shows predominant expression in non-mitotic cells of somatic tissue, while EZH2 is primarily expressed in proliferating cells. The role of EZH2 is critical in the regulation of diverse biological processes, such as tumor angiogenesis, cell apoptosis, EMT, as well as cell migration and invasion [33–35] (Fig. 2).

The binding affinity of EZH2 to histone substrates exhibits an inverse correlation with the degree of H3K27 methylation [36]. Within the multisubunit core region of PRC2, the enzymatic EZH2-PRC2 reaction takes place, regulating the spatial morphology of PRC2 and activating it through the interaction mechanism involving EZH2, EED, and H3K27me3. When the N-terminus of EZH2 binds to the targeted region of EED, an α-helix is formed, and the WD40 repeat sequence of EED recognizes PRC2-EZH2 deposited on the same or adjacent nucleosomes with H3K27me3, thereby promoting EZH2 activation [37, 38]. PRC2 can also trimethylate cofactors JARID2 and PALI1, mimicking H3K27me3 and binding to EED to generate allosteric stimuli. During this process, the EZH2 CXC domain binds to DNA, positioning the H3 tail at the EZH2 catalytic center [39–41]. In human HNSCC cells, the absence of EZH2 partially impairs both in vitro and in vivo proliferation and invasion abilities. However, the specific details regarding whether and how EZH2 targets and induces cell apoptosis are still unclear, suggesting a direction for future research development.

## Mechanisms of PRC2 recruitment to the chromatin

Since EZH2 is not a DNA-binding protein but rather acts as a chromatin regulatory factor, its recruitment to specific genomic sites necessitates the involvement of various cofactors and chromatin characteristics. These can be broadly categorized into four groups: histone modifiers, site-specific DNA-binding proteins, RNA molecules, and CGIs [42, 43].

## Histone modifiers

Apart from the aforementioned core subunit of PRC1 and the six ring finger proteins (PCGF1-6) mentioned earlier, they correspond to the subcomplexes PRC1.1-1.6, respectively. Within these subcomplexes, there is a CBX subunit that specifically binds to H3K27me3 and is known as typical PRC1 (cPRC1). However, cPRC1 only interacts with RYBP, RING1, and Yin Yang 1 (YY1) binding proteins, which do not recognize the variant PRC1 (vPRC1) associated with H3K27me3. PRC1 catalyzes the monoubiquitination of histone H2A at lysine 119 (H2AK119ub1) through the RING1 protein. This modification is recognized by JARID2, leading to its recruitment to chromatin by PRC2 [44, 45]. Recent studies have demonstrated that the vPRC1 form is capable of sensing the deposition of H2AK119ub1, which contributes to its stability, particularly at highly enriched modification sites where H2AK119ub1 deposition plays a pivotal role in establishing PcG-mediated repression mechanisms to maintain cellular transcriptional identity [46].

## Site-specific DNA binding proteins

EZH2-mediated transcription factors facilitate the recognition and subsequent recruitment of specific protein sequences encoded in DNA by PRC2. The methylation reading is further enhanced when AEBP2, a component of PRC2.2, is added to RBBP4-PRC2, as demonstrated in vitro studies. Recent research has confirmed this stimulatory effect, indicating that AEBP2 promotes increased binding to oligonucleosomes and enhances the catalytic activity of both EZH1-PRC2 and EZH2-PRC2. Interestingly, the mechanism underlying this stimulation differs from the allosteric stimulation observed in the interaction between H3K27me3 and EED. JARID2, another subunit of PRC2.2, is believed to act as a potential recruiter and modulator of PRC2 activity. This belief is supported by its interaction with PRC2, significant overlap in genomic targets, and moderate affinity for GC-rich DNA through its ARID domain. Although the loss of JARID2 leads to differentiation defects in mESCs, it only has a modest impact on PRC2 binding and H3K27me3 patterns [47–49].

## RNA molecules

Numerous studies have extensively investigated the interaction between small RNAs and PRC2. For instance, the long non-coding RNA HOTAIR, which is transcribed from the Homeobox C gene (HOXC) site, exerts various functions in different malignant tumors [50, 51]. However, there is an ongoing debate regarding whether long non-coding RNAs recruit PRC2 to chromatin and participate in the repression of HOXD loci mediated by HOTAIR. Moreover, RNA immunoprecipitation experiments have revealed that EZH2 or SUZ12 can bind to a substantial amount of "non-specific" RNA. The proposal suggests that short transcripts originating from genes bound by PRC2 could potentially function as a mechanism to anchor PRC2 to the promoters of their target genes. MicroRNAs play a vital role in the regulation of PRC. For instance, the functionality of PRC2 complex relies on the presence of SUZ12. Elevated levels of SUZ12 have been linked to the development of cancer stem cells, whereas restoration of miR-128 expression hinders PRC activity. Mir-128-mediated downregulation of PRC components leads to a significant repression of PRC-dependent histone modifications, reduced expression of CD133, and decreased tumor suppressor p21 levels. These findings underscore the significance of microRNAs as molecules capable of targeting the entire pathway by concurrently inhibiting multiple components, thereby impeding their redundant role in tumors [52]. Nevertheless, further investigation is required to elucidate the specific mechanism underlying this model.

**Fig. 3:**
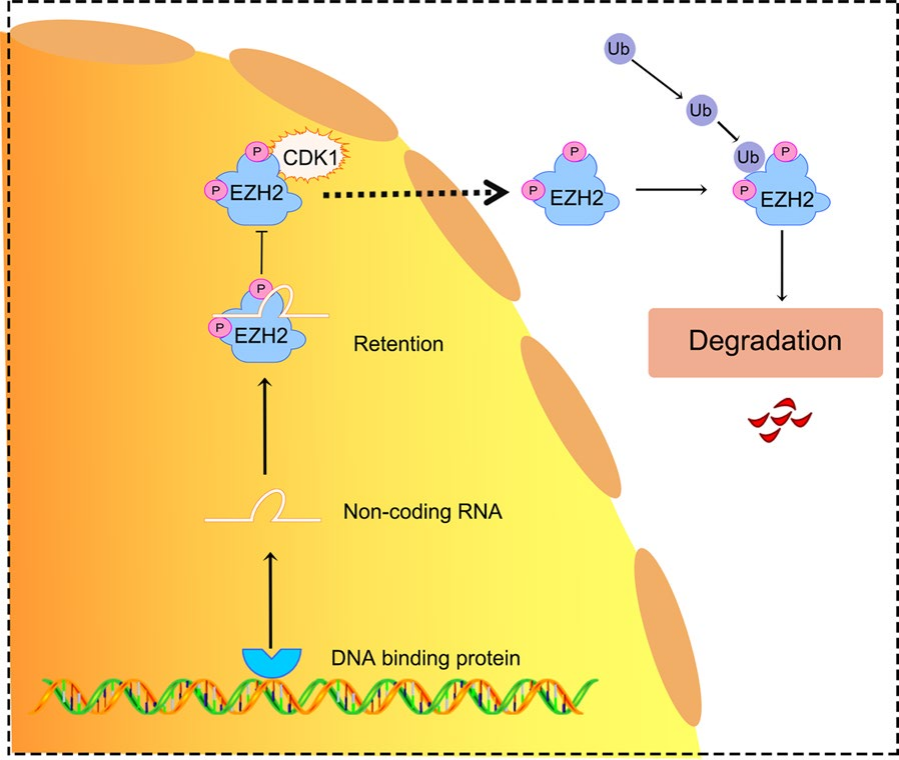
Inducing small molecule non-coding RNAs to block CDK1-mediated EZH2 degradation of tumors in HNSCC. Transcription factors, which are DNA-binding proteins, play a crucial role in activating small non-coding RNAs and directly interacting with EZH2 within the nucleus of HNSCC. This specific interaction acts as an inhibitor, preventing the CDK1-mediated phosphorylation of EZH2 at the T345 and T487 sites. Furthermore, the presence of small non-coding RNAs effectively hinders the translocation of EZH2 from the nucleus to the cytoplasm, consequently resulting in ubiquitination and subsequent degradation of EZH2 by the proteasome [110]

Some researchers propose that RNA competes or interferes with the chromatin binding interface, thereby preventing PRC2 from binding to active transcriptional regions. This viewpoint is backed by foundational experiments demonstrating the competition between RNA and DNA for PRC2 binding, while the autocatalytic activity of PRC2 remains unaffected in the presence of RNA. Despite these controversies, it is clear that EZH2 is an RNA-binding protein and RNA plays a vital role as a regulatory factor in the global localization of EZH2 and PRC2 on the genome [53–55] (Fig. 3).

## CGIs

CGIs are DNA sequences that span hundreds of base pairs and are distinguished by having a high frequency of CpG dinucleotides. The recruitment of PRC2 shares similarities with TF-mediated gene regulation, wherein transcription factors use sequence-specific DNA binding domains to target genomic regions and recruit cofactors through protein–protein interaction domains. While sequence-specific factors may not be directly involved in recruitment as CGIs lack repetitive motifs, PRC2 can still bind to CGIs through synthetic or non-evolutionary sequences. In contrast, CGIs that are bound by PRC2 possess a high CpG content, distinct DNA conformation, and are characterized by the absence of transcriptional activity and co-occupancy with PRC1 and H2AK119ub1. Therefore, based on these characteristics, PRC2 subunits or interactors that persist on CGIs for prolonged periods can be considered as recruiters of PRC2 [56, 57].

## EZH2/PRC2 participates in the epigenetic regulation of HNSCC

In addition to genetic changes, cancer cells often employ chromatin-based mechanisms to epigenetically suppress innate immune responses and tumor immunogenicity. Compared to normal mucosa or adjacent normal tissues, 50–60% of HNSCC tissues exhibit overexpression of EZH2, which is closely associated with tumor differentiation status. Furthermore, EZH2 can induce malignant transformation of oral leukoplakia and epithelial–mesenchymal transition in HNSCC [58, 59]. Studies have also demonstrated that upregulation of EZH2 is linked to tumor invasiveness and poor prognosis in HNSCC [60]. Previous studies in HNSCC indicate a correlation between the expression of EZH2 and its specific location. Overexpression is more prevalent in oropharyngeal and oral cancers compared to laryngeal cancer. For instance, Kim et al. reported that the expression level of EZH2 in oropharyngeal cancer was twice as high as in all other head and neck cancers (41% vs. 24%) [61]. Other studies have described the expression rate of EZH2 in oral tongue squamous cell carcinoma as high as 97%. However, Nienstedt's study found a relatively low percentage of EZH2 expression in tongue cancer (6%) compared to other cohorts (39–100%), indicating potential differences in the molecular environment among different mucosal regions of head and neck cancer that could influence EZH2 [62, 63].

**Fig. 4:**
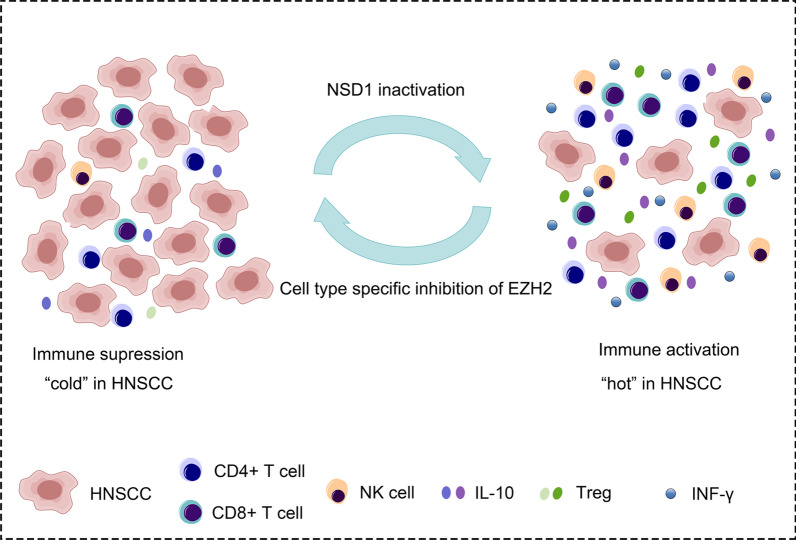
EZH2 inhibitors and tumor immune effects. The inactivation of NSD1 (the decisive H3K36me2 catalytic enzyme in embryonic stem cells) results in transcriptional suppression of innate immune genes and decreased infiltration of immune cells into tumors by deleting H3K36me2 and increasing H3K27me3. Inhibiting EZH2 can reactivate the interferon response, restore immune cell infiltration, and impede the growth of NSD1 mutant SCC [111]. EZH2 inhibitors enhance the activity of CD8 T effector cells, stimulate the secretion of chemokines by tumor-infiltrating DC cells, and facilitate the trafficking of effector T cells to the tumor microenvironment (TME). They also decrease the stability of the Treg lineage, impair its function, and partially overcome immune checkpoint inhibitor resistance. Additionally, EZH2 inhibitors promote the maturation and activation of NK cells, as well as increase the expression of activating receptors, thus bolstering innate anti-tumor immunity [112]

In Li et al.'s study, it was observed that innate immune genes in HNSCC cells were particularly affected by the imbalance of H3K36me2 and H3K27me3. Sequencing analysis revealed an enrichment of H3K27me3 at the gene promoter binding sites of interferon-regulated transcription factors, such as IRF8 and MYB, in cells with NSD1 knockout. This suggests that NSD1 might play a role in regulating the recruitment of interferon-regulated transcription factors in epithelial tissue, preventing PRC2-mediated silencing, and maintaining the expression of interferon-responsive genes [64, 65] (Fig. 4). The EZH2/STAT3 axis predominantly functions as a carcinogenic pathway, where EZH2 plays a crucial role in enhancing STAT3 activity through methylation. For instance, NF-YA, a transcription regulator, can induce the overexpression of EZH2, resulting in the augmentation of STAT3 activity via lysine methylation. This, in turn, leads to the upregulation of VEGF expression and facilitates angiogenesis in black HNSCC. Furthermore, EZH2 can also promote STAT3 phosphorylation at pY705 through independent mechanisms unrelated to STAT3 methylation. The current body of research indicates that tumor glycolysis significantly influences EMT invasion and various types of cancer. Most cancer cells employ glycolysis as a means to generate energy for rapid growth and metastasis. In HNSCC, increased EZH2 expression corresponds to enhanced glycolysis, EMT, migration, invasion, as well as heightened levels of STAT3 phosphorylation and reduced fox01 expression. Some scholars propose that EZH2 can regulate N-cadherin and vimentin expression at both the mRNA and protein levels, while simultaneously suppressing E-cadherin expression, thereby promoting migration and invasion of OSCC cells [66]. In addition, recent findings suggest that the role of EZH2 in certain tumors differs from its role in HNSCC. Specifically, in ovarian cancer cells, phosphorylation of the threonine 372 site on EZH2 inhibits proliferation and migration in vitro by binding to STAT3 and reducing the levels of pSTAT3. Moreover, this phosphorylation also suppresses the growth of ovarian xenograft tumors in vivo. In contrast, upregulation of EZH2 in HNSCC leads to a significant increase in the expression of tyrosine phosphorylated STAT3 at site 705, while the overall expression of STAT3 remains unchanged. Consequently, EZH2 may regulate HNSCC cell invasion and tumor glycolysis through the activation of STAT3. The observed differences in EZH2's function may be attributed to variations in the cancer microenvironment [68].

HOTAIR, a long non-coding RNA encoded by the HOXC locus, serves as an oncogenic gene and has diverse roles in different malignant tumors. It acts by recruiting EZH2 to catalyze H3K27 trimethylation, leading to the suppression of downstream tumor suppressor genes. In glioblastoma, HOTAIR influences cell cycle progression and invasion by activating the catenin signaling pathway. By targeting HOTAIR and EZH2, it is possible to induce apoptotic pathways related to mitochondria, thereby inhibiting the biological behavior of head and HNSCC [70]. Additional experiments have shown that EZH2 also impacts the granulocyte-associated cell death pathway. Inhibiting EZH2 can trigger the accumulation of Ca2 + in cells, leading to mitochondrial membrane potential (ΔΨM) loss, G1 phase cell cycle arrest, and changes in mitochondrial-related cell death pathway proteins. These changes indicate that MCU (mitochondrial calcium uniporter) is a highly selective channel with calcium transport ability [71]. MICU1 has been shown to regulate mitochondrial calcium single transporter proteins, pumping calcium from the cytoplasm into mitochondria [70]. In the absence of MICU1, mitochondria selectively load calcium, resulting in excessive production of reactive oxygen species and sensitivity to apoptotic stress. Targeting MICU1 can compensate for the role of EZH2 in regulating tumor cell apoptosis [72].

There is supporting evidence regarding the biological behavior of HNSCC, indicating that downregulation of EZH2 can inhibit proliferation and activate apoptosis in HNSCC cells. A study by Cao et al. confirmed a biological connection between EZH2 overexpression and the precursor state of HNSCC, oral leukoplakia, as well as its involvement in early-stage oral tumor development by promoting cell cycle progression [72]. Additionally, decreased expression of EZH2 significantly reduces migration and invasion rates in knocked-down OSCC (oral squamous cell carcinoma) cells [73].

### Therapeutic targeting of EZH2 in HNSCC

Currently, platinum-based regimens (platinum, 5-FU, and cetuximab) are the standard treatment for HNSCC. However, the most important task at present is to identify treatment targets that can enhance the anti-tumor effect of standard treatment and improve the prognosis for HNSCC patients [74]. In recent times, chromatin dysregulation has emerged as a promising target for molecular markers and therapeutic interventions in cancer. While chromatin modifying enzyme inhibitors have been approved for the treatment of hematological and soft tissue malignancies, their efficacy in tumor immunotherapy, particularly in the context of complex genomic HNSCC, still lacks sufficient evidence[ 75].

In HNSCC, the activity of EZH2 may be enhanced by phosphorylation at the serine 21 site, which leads to direct binding and methylation of signal transducer and activator of transcription factor 3 (STAT3). Aberrant activation of STAT3 is commonly observed in 70% of cancer types and is correlated with unfavorable prognosis in patients. Therefore, targeting STAT3 represents a potential therapeutic strategy for HNSCC [76, 77]. Furthermore, the precise mechanism by which EZH2 and STAT3 interact remains unclear; nevertheless, EZH2 has been found to facilitate the oncogenic function of STAT3 by demethylating K49 [78]. According to the study conducted by Sun et al., targeting the STAT3/HOTAIR/EZH2 axis could present a novel therapeutic approach for effectively treating PI3K-activated HNSCC patients with a combination of cisplatin and cetuximab [79].

In addition, the regulation of microRNAs is also implicated in this process. Manipulating the activity of STAT3 signaling, either by weakening or enhancing it using STAT3 plasmids, alters the state of the EZH2/miR-200 axis, thereby modulating the invasion and migration of cell lines associated with HNSCC. Moreover, disrupting the STAT3/EZH2/miR-200 axis results in changes to both f-actin morphology and the expression of markers related to epithelial–mesenchymal transition. It should be noted that the oncogenic role of STAT3 in HNSCC is compromised when EZH2 function is lost [80–82]. While it is still in its early phases, the findings thus far are promising and indicate that targeting STAT3 holds promise as a potential therapeutic approach.

Conversely, Wnt/β-Catenin has been implicated in the build-up of β-Catenin and EZH2 within cisplatin-resistant and cancer stem cell (CSC) populations. The upstream genes APC and GSK3, which are involved in the β-Catenin signaling pathway, exhibit reduced activity, whereas the downstream gene MMP7 shows increased expression. Effective reduction of both the in vitro CSC population and in vivo tumor volume can be achieved through combined inhibition of β-Catenin and EZH2. Notably, suppressing EZH2 boosts the expression of APC and GSK3β, while inhibiting Wnt/β-Catenin decreases MMP7 levels [83, 84]. By sensitizing chemotherapy-resistant cells to cisplatin, inhibitors of EZH2 and β-Catenin pave the way for improved therapeutic outcomes. Furthermore, the binding of EZH2 and H3K27me3 to the promoter of APC leads to its inhibition, thereby contributing to the accumulation of tumor stem cells and the development of chemotherapy resistance [63, 85–87]. Furthermore, long non-coding RNA H19 has been found to be regulated by EZH2 in squamous cell carcinoma of the tongue, promoting the occurrence and progression of tumors by influencing β-Catenin and GSK3β gene expression [88].

Currently, there are varying opinions on the mechanism of cisplatin resistance. The anticancer effect of cisplatin is achieved through the formation of covalent platinum DNA adducts following DNA damage. To enhance cisplatin response in HNSCC cells inhibited by EZH2, the following hypothesis has been proposed: H3K27 methylation, mediated by EZH2, serves as a marker of heterochromatin, which prevents external factors from accessing densely packed DNA. Consequently, inhibiting EZH2 results in loss of chromatin compaction, potentially facilitating drug-DNA interactions, DNA damage, and subsequent cancer cell death [89, 90]. Tazemetostat, the first therapy specifically approved for the treatment of epithelioid sarcoma in the USA, has garnered significant attention due to its potential [91]. A recent investigation conducted by Liu et al. examined the potential of using tazemetostat, an EZH2 inhibitor, and sunitinib, an MDSC inhibitor, as therapeutic strategies to overcome immune evasion in HNSCC [92]. The application of tazemetostat was found to enhance the release of tumor antigens and facilitate antigen processing and extraction, resulting in increased infiltration of cytotoxic T lymphocytes. However, it should be noted that the effectiveness of sunitinib is impeded by elevated levels of myeloid-derived suppressor cells (MDSCs) within the tumor microenvironment, which are induced by both tumor burden and the administration of EZH2 inhibitors [93, 94].

In oncology research, the use of immune checkpoint blockade (ICB) drugs has garnered significant interest as a tumor immunotherapy strategy. Nevertheless, there remain numerous unresolved inquiries and a challenging path forward in the case of HNSCC. For example, the expected response was not observed when targeting EZH2 in solid tumors. Interestingly, EZH2 inhibitors resulted in an elevation of MDSCs within the tumor microenvironment [95]. This inconsistency could potentially be attributed to the specific immune landscapes found within tumors, which subsequently impact their responsiveness to diverse immunotherapy approaches [96, 97]. In the realm of targeted drug research, EZH2 shows great potential as a therapeutic target for oral squamous cell carcinoma. Thus, it is crucial to investigate the impact of EZH2 deficiency in models more closely resembling HNSCC and consider disease stages within these models. Currently, EZH2 inhibitors such as tazemetostat, GSK2816126, and PCI-1205 are being evaluated in clinical trials for other diseases like lymphoma and medulloblastoma, necessitating further investigation [98, 99]. The regulation of epigenetics is a complex process involving multiple functional groups. For the proper execution of its function, EZH2, serving as the catalytic core subunit of PRC2, necessitates interaction with EED and SUZ12. Therefore, the disruption of PRC2's structure or the inhibition of EED or SUZ12 can indirectly impede EZH2, presenting novel prospects for inhibitor development. Recently, targeted chimeras (PROTACs) have emerged as a promising approach in drug development. These PROTACs, specifically PROTAC EED hypoglycemic agent-1 and PROTAC EED hypoglycemic agent-2, combine two active compounds with inhibitory properties against PRC2. They effectively bind to EED, resulting in the functional suppression of PRC2 and consequently hindering proliferation in vitro of EZH2 mutant cell lines [100].

Table 1 provides a summary of commonly used EZH2/PRC2 related inhibitors and their respective targets of action. In the past decade, researchers have developed several inhibitors targeting EZH2, an important protein involved in cancer progression. One of these inhibitors is 3-Deazaneplanocin A (DZNep), which functions by inhibiting S-adenosylhomocysteine (SAH) hydrolase. This inhibition leads to the accumulation of SAH, a byproduct resulting from the transfer of methyl groups from S-adenosyl-methionine (SAM). The buildup of SAH prevents further SAM-mediated methyl transfer. Notably, DZNep has been found to induce cell cycle genes and apoptosis in primary AML cells [107]. Additionally, other more specific inhibitors have been investigated, such as GSK126 and EI1, which have been tested in cell culture or xenograft models. GSK126, a small molecule inhibitor, competitively binds to SAM and exhibits high selectivity for EZH2 over other human methyltransferases (~ 0.5 nM Ki value compared to 20 other human methyltransferases) [113]. On the other hand, UNC1999 is an orally bioavailable inhibitor that selectively targets both EZH1 and EZH2, with a tenfold preference for EZH2 over EZH1 (compared to > 150-fold preference with GSK126). In preclinical models of MLL-rearranged leukemia, UNC1999 has shown the ability to suppress tumor growth [124]. Another promising candidate is Valemetostat Tosylate (DS-3201b), a dual inhibitor of EZH1 and EZH2. It has demonstrated synthetic lethality in malignancies that overexpress EZH2 or have mutations in histone-modifying genes, as observed in preclinical models [125]. These advancements in developing EZH2 inhibitors provide potential therapeutic strategies for various cancer types, offering new avenues for targeted treatment and potentially improving patient outcomes.

**Table 1:** Common EZH2 inhibitors

Inhibitors of EZH2				
Name	Function	Results	Cancer	References
3-deazaneplanocin A (DZNep)	Via mitochondria-dependent cell death	Apoptosis	HNSCC	[106]
GSK-126	Decreases the invasion of prostate cancer stem/progenitor cells	Decreases the invasion	Prostatic cancer (PCa)	[112]
GSK926/GSK343	Inhibitor of EZH2	Inhibit cell proliferation	Breast cancer cells and PCa	[113]
ASC-J9® (a novel androgen receptor degradation enhancer)	S newly developed AR degradation enhancer with the unique capability to selectively degrade AR protein in some cell types	Decreases the invasion	PCa	[112]
EPZ5687	SAM-competitive inhibitor	Prolong G1 phase, shorten S phase and G2/M phase	Lymphoma	[114]
EPZ011989	Potent and selective EZH2 inhibitor	Inhibiting tumor growth	Lymphoma	[115]
Tazemetostat	Enhancer of zeste homolog 2 protein inhibitors	Antitumor activity	Epithelioid sarcoma (ES)	[91]
EBI-2511	A scaffold based on tazemetostat	Antitumor activity	NHL	[116]
CPI-169	Potent and selective EZH2 inhibitor	Inhibit the activity of EZH2	NHL	[117]
Lirametostat (CPI-1205)	Inhibitor of EZH1 and EZH2	Apoptosis	Multiple myeloma and plasmacytoma	[118]
ZLD10A	Potent and selective EZH2 inhibitor	Apoptosis	NHL	[119]
JQEZ5	Inhibitor of EZH2	Inhibition of cell colony production	Chronic myeloid leukemia (CML)	[120]
PF-06726304	Inhibitor of EZH2	Inhibit cell proliferation	Lymphoma	[121]
EZH2-IN-3	Inhibitor of EZH1 and EZH2	Inhibit cell proliferation	Lymphoma	[122]
UNC1999	Inhibitor of EZH1 and EZH2	Induce autophagy	Lymphoma	[123]
Valemetostat (DS-3201, DS3201b)	Inhibitor of EZH1 and EZH2	Restore immune function	T-cell leukemia lymphoma (ATL)	[124]
(R)-ORS1	SAM-competitive inhibitor	Inhibiting tumor growth	Lymphoma	[125]
PF-06821497	Inhibitor of EZH2	Inhibiting tumor growth	Lymphoma	[126]
Oxetinib (AZD9291)	EGFR mutant inhibitor	Impaired PRC2 function	Lymphoma	[127]

## EZH2/PRC2 mediates EMT process in HNSCC

The main characteristic of benign lesions transitioning to metastatic cancer is their ability to overcome intercellular adhesion and invade surrounding tissues, and this process is largely driven by epithelial–mesenchymal transition (EMT). In EMT, the absence of e-calmodulin and upregulation of mesenchymal markers are key molecular features [101]. Regarding the effects of EMT-related molecules, silencing of EZH2 leads to increased expression of E-cadherin, reduced expression of N-cadherin and Vimentin, without altering the signal transduction of Snail/Slug. This suggests that the regulation of E-cadherin and induction of EMT in cells are not solely dependent on Snail/Slug. Furthermore, EZH2 introduces H2K27me3 into the E-cadherin gene promoter, specifically inhibiting E-cadherin in a snail/slug-independent manner. Consequently, there is an increase in cell migration and invasion, as observed in previous studies [102–104].

**Fig. 5:**
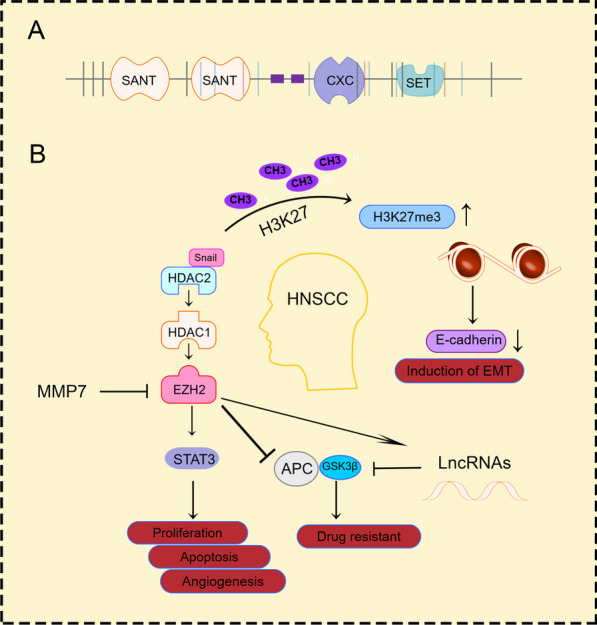
**A** EZH2 regulates the function of HNSCC. A Molecular structure of EZH2 (UniProt, 751 amino acids, isoform a). SANT: SANT SWI3, ADA2, N-CoR and TFIIIB” DNA-binding domain, CXC: Tesmin/TSOl-like CXC domain, SET: Su (var)3–9, Enhancer-of-zeste, Trithorax.** B** EZH2 serves as an upstream mediator of non-coding RNAs expression in HNSCC and is associated with the STAT3 pathway. Due to its transcriptional role, this pathway can target miRNAs to affect cancer cell proliferation, drug resistance, EMT process, and invasion, thereby determining cancer cell response to treatment

One of the common mutated genes in human cancer is FAT1. Recent research by Pastushenko et al. has revealed the molecular mechanism phenotype of EMT induced by the loss of cell polarity and adhesion caused by Fat1 deficiency. Interestingly, a significant decrease in the overall level of H3K27me3 was observed in FAT1 knocked out cells, indicating that EZH2 may play a role in downregulating the functional loss of FAT1 mutant activity. Conversely, treatment with inhibitors led to an increase in H3K27me3 levels in FAT1 knocked out cells, supporting the view that CAMK2 activation can inhibit EZH2 and PRC2 activity in tumor cells. In summary, the lack of FAT1 promotes EZH2 inactivation, affecting the EMT process in tumor cells, coordinating epigenetic changes, and maintaining the epithelial state. These findings highlight the significant impact of this process [105]. Additional investigations have also exhibited that in HNSCC, EZH2 can obstruct EMT processes both in vitro and in vivo by controlling downstream signaling pathways, including the STAT3/vascular endothelial growth factor receptor 2 (VEGFR2) axis. The suppression of EZH2 results in the downregulation of critical molecules and markers associated with EMT along the STAT3/VEGFR2 axis, while concurrently bolstering the expression of E-cadherin in HNSCC cells. The deliberate targeting of the EZH2/STAT3/VEGFR2 axis proves to be an effective approach in diminishing the mobility of HNSCC cells [106]. In HNSCC, the expression of HOTAIR contributes to increased cancer cell invasion by repressing E-cadherin transcription and inducing EMT [107]. Multiple investigations have indicated that EZH2 is involved in promoting the progression of HNSCC and EMT through its regulation of STAT3 or VEGFR2. This mechanism, which involves EZH2, holds promise as a potential avenue for the development of strategies aimed at effectively managing the progression and metastasis of HNSCC by targeting EZH2/PRC2 (Fig. 5).

### Conclusions/future directions

The phenotypic characteristics and mechanisms of PcG proteins and complexes underscore their physiological significance and their potential in targeted therapy. These intricate and precise protein structures dictate their specific functions, and comprehending their environmental specificity and genetic alterations in tumors is vital for developing effective therapeutic approaches. Current drugs have been developed to target core subunits of PRC2 and PRC1, such as EZH2, and understanding the specific roles of individual complex variants in particular cancer types is pivotal [108].

In HNSCC, extensive scientific research has greatly enhanced our understanding of the structure and biological functions of the PRC2 complex. Through the combination of biochemical and proteomic analyses, numerous proteins associated with EZH2 have been identified up to now. Our thorough investigation into the activity and functionality of PRC2, coupled with advancements in cutting-edge techniques like gene editing, stem cell biology, and single-cell transcriptome analysis, will contribute to the development of new inhibitors and therapeutic targets.

Despite advancements in research, the prognosis for HNSCC remains suboptimal. The integration of novel treatment strategies, targeted therapies, and non-invasive, highly specific biomarkers has undoubtedly improved the survival rates of HNSCC patients. Yet, numerous challenges and unresolved issues persist. First and foremost, while the pivotal role of EZH2/PRC2 in promoting and counteracting cancer in HNSCC has been extensively investigated, further elucidation is needed regarding the regulation of its diverse expression mechanisms in HNSCC patients across different clinical scenarios. This understanding would offer valuable insights for early tumor detection and treatment. Secondly, the unique specificity of EZH2 in HNSCC tissues, which can induce malignant transformation of oral leukoplakia and EMT processes, holds paramount importance in comprehending the initiation and progression of HNSCC. However, the specific mechanisms and influencing factors underlying this phenomenon require further exploration. Lastly, preliminary research findings show promise in targeting STAT3 as a potential therapeutic approach when the carcinogenic effect of EZH2/STAT3 in HNSCC is compromised. Nonetheless, molecular targeted therapy presents its own set of challenges while remaining an avenue filled with hope and potential.

While EZH2 and PRC2 activation in HNSCC have been well-documented, the regulation of these distinct activities of polycomb proteins remains poorly understood. The involvement of cofactors and the alterations in PRC binding during differentiation vary among different cell states, resulting in a lack of consensus. Moreover, the specific components within the tumor microenvironment that contribute to the divergent biological behavior mediated by EZH2/PRC2 in HNSCC compared to other solid tumors are not yet identified. Furthermore, the intricate interaction between RNA and PRC appears to be multifaceted, leading to contradictory findings that warrant further investigation. In summary, the epigenetic regulation of EZH2/PRC2 plays an important role in the progression of HNSCC and has clinical significance as a risk predictor for this disease and in molecular targeted therapy.

## Data Availability

Not applicable.
